# Surgical outcomes of DeBakey type I and type II acute aortic dissection: a propensity score-matched analysis in 599 patients

**DOI:** 10.1186/s13019-021-01594-9

**Published:** 2021-07-30

**Authors:** Chun-Yu Lin, Tao-Hsin Tung, Meng-Yu Wu, Chi-Nan Tseng, Feng-Chun Tsai

**Affiliations:** 1 Department of Medicine, College of Medicine, Chang Gung University, Taoyuan City, Taiwan; 2 Department of Cardiothoracic and Vascular Surgery, New Taipei Municipal TuCheng Hospital, No.6, Sec.2, JinCheng Rd, TuCheng, New Taipei City, 236 Taiwan; 3 Evidence-Based Medicine Center, Taizhou Hoispital of Zhejiang Province Affiliated To Wenzhou Medical University, Linhai, Zhejiang China; 4 Department of Cardiothoracic and Vascular Surgery, Chang Gung Memorial Hospital, Linkou Medical Center, Taoyuan City, Taiwan

**Keywords:** Acute type A aortic dissection, DeBakey classification, Propensity score-matched analysis, Survival, Reintervention

## Abstract

**Background:**

The DeBakey classification divides Stanford acute type A aortic dissection (ATAAD) into DeBakey type I (D1) and type II (D2) according to the extent of acute aortic dissection (AAD). This retrospective study aimed to compare the early and late outcomes of D1-AAD and D2-AAD through a propensity score-matched analysis.

**Methods:**

Between January 2009 and April 2020, 599 consecutive patients underwent ATAAD repair at our institution, and were dichotomized into D1 (n = 543; 90.7%) and D2 (n = 56; 9.3%) groups. Propensity scoring was performed with a 1:1 ratio, resulting in a matched cohort of 56 patients per group. The clinical features, postoperative complications, 5-year cumulative survival and freedom from reoperation rates were compared.

**Results:**

In the overall cohort, the D1 group had a lower rate of preoperative shock and more aortic arch replacement with longer cardiopulmonary bypass time. The D1 group had a higher in-hospital mortality rate than the D2 group in overall (15.8% vs 5.4%; *P* = 0.036) and matched cohorts (19.6% vs 5.4%; *P* = 0.022). For patients that survived to discharge, the D1 and D2 groups demonstrated similar 5-year survival rates in overall (77.0% vs 85.2%; *P* = 0.378) and matched cohorts (79.1% vs 85.2%; *P* = 0.425). The 5-year freedom from reoperation rates for D1 and D2 groups were 80.0% and 97.1% in overall cohort (*P* = 0.011), and 93.6% and 97.1% in matched cohort (*P* = 0.474), respectively.

**Conclusions:**

Patients with D1-AAD had a higher risk of in-hospital mortality than those with D2-AAD. However, for patients who survived to discharge, the 5-year survival rates were comparable between both groups.

## Introduction

Acute aortic dissection (AAD) is a cardiovascular emergency associated with high morbidity and mortality rates [1, 2]. The Stanford and DeBakey classifications are commonly used to determine the extent of the dissected aortic segment [3, 4]. Stanford acute type A aortic dissection (ATAAD) requires prompt surgical treatment for life-saving and accounts for 62%–67% of the entire AAD population according to the International Registry of Acute Aortic Dissection database [2, 5]. ATAAD can be divided into DeBakey type I (D1) and type II (D2) based on whether the dissected aorta is confined to the ascending portion only or extends to the aortic arch and descending aorta. In previous studies, D1-AAD was usually associated with inferior survival rates and a higher aortic reintervention probability compared to D2-AAD [6, 7]. However, disparities in regard to clinical presentation, preoperative condition, and aortic repair procedures commonly exist between patients with D1-AAD and D2-AAD owing to the different complexities of aortic anatomy and involved end-organs. Therefore, some bias among patient selection might have affected the reliability of previous outcome analyses. In the present study, we performed a retrospective propensity score-matched analysis of the database from an individual aortic surgery center and compared the individual characteristics and early and late outcomes of patients who underwent surgical repair for D1-AAD and D2-AAD.

## Materials and methods

### Patient enrollment and preoperative management

**Fig. 1:**
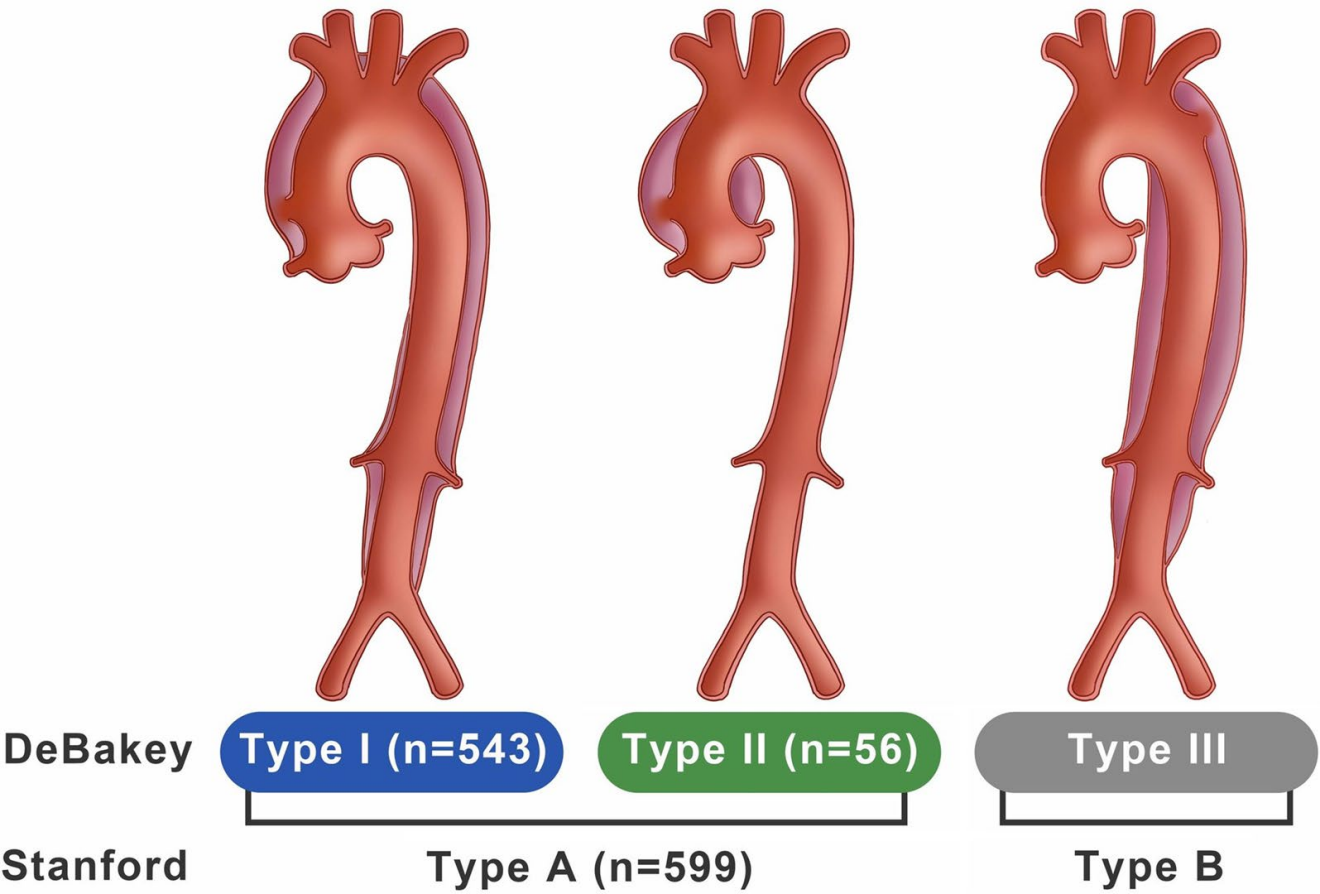
Distribution of ATAAD patients from January 2009 to April 2020. ATAAD, acute type A aortic dissection

**Fig. 2:**
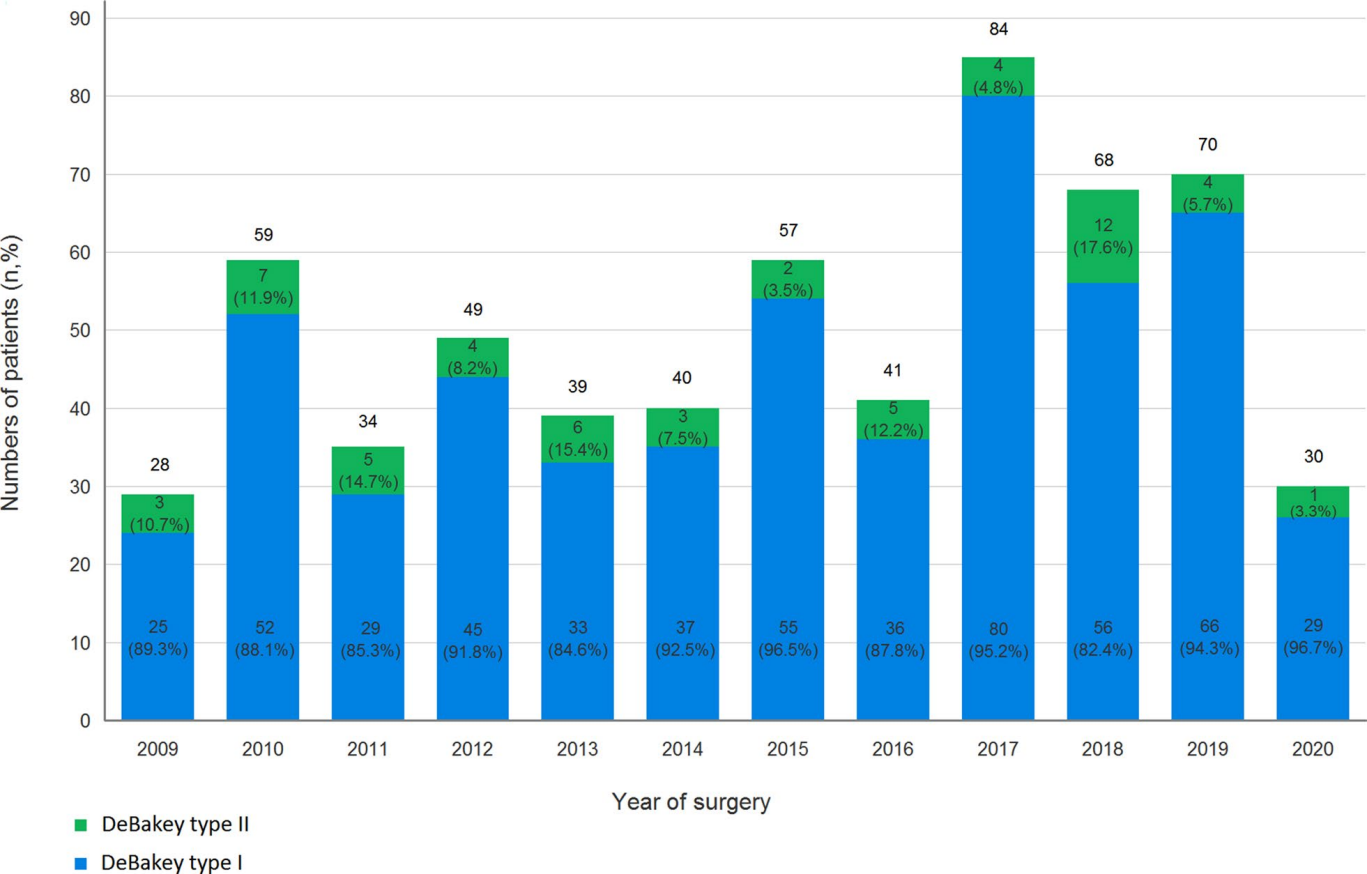
Annual cases in the overall cohort, DeBakey type I group, and DeBakey type II group during the study period

The study protocol was conducted by the approval of the Institutional Review Board of Chang Gung Medical Foundation (approval number 202001566B0). The requirement for informed consent was waived due to the retrospective nature of the study. A total of 599 consecutive adult patients underwent emergency ATAAD repair at this institution between January 2009 and April 2020. All patients were diagnosed via helical computed tomography in the emergency department, and the extent of aortic dissection was analyzed. The 599 included patients were dichotomized into D1 (n = 543; 90.7%) and D2 groups (n = 56; 9.3%) based on the DeBakey classification (Fig. 1). The annual cases of the overall cohort, D1, and D2 groups during the study period are illustrated in Fig. 2. When the diagnosis of ATAAD was confirmed, patients were emergently transferred to the operating room within 30 min. If patients presented with hypertension or tachycardia before surgery, their hemodynamics were stabilized with intravenous beta-blockers to maintain systolic blood pressure (SBP) < 120 mmHg and a heart rate of 60–70 bpm, according to the established guidelines [8]. If patients presented with shock status, medical resuscitation and surgical rescue procedures were applied according to the standardized protocols previously reported by this institute [9, 10].

### ATAAD repair procedures and postoperative care

The technical aspects of aortic repair procedures were detailed in previous studies [11, 12]. Double artery cannulation using a combination of right axillary and femoral arterial access and the antegrade cerebral perfusion (ACP) strategy were preferably implemented for patients who presented with relatively stable preoperative condition. Otherwise, isolated femoral artery cannulation with retrograde cerebral perfusion (RCP) was preferred for patients with unstable hemodynamics. Following sternotomy, the right atrium or vena cava was cannulated and cardiopulmonary bypass (CPB) with deep hypothermia was initiated. In general, the dissected aorta was replaced with a Dacron prosthetic graft based on the location of the major entry tear and preoperative presentation. The tubular ascending aorta (AsAo) was routinely replaced with aortic valve resuspension, and the proximal anastomosis was usually performed first, followed by open distal anastomosis under circulatory arrest. All graft-aorta anastomoses were reinforced with Teflon felt. During circulatory arrest, the femoral arterial flow was temporarily suspended and selective ACP through the right axillary artery or RCP through the superior vena cava was performed. Concomitant aortic root replacement with a composite Valsalva graft was performed if the extent of aortic dissection involved the aortic root and was complicated with severe aortic regurgitation. After undergoing ATAAD repair, all patients were transferred to a specialized cardiovascular intensive care unit for further treatment and observation. The ventilator-weaning protocol was initiated at 12–24 h after surgery if unstable hemodynamics, persistent arrhythmia, signs of organ malperfusion, and active bleeding were not observed.

### Statistical analyses

Statistical analyses were performed using SPSS for Windows (version 22.0; IBM Corp., Armonk, NY, USA). Data are presented as means ± standard deviation for numerical variables and as numbers and percentages for categorical variables. To compare the intergroup disparities between the D1 and D2 groups, we used the independent t-test for numerical variables and the chi-square or Fisher’s exact test for categorical variables, respectively. A propensity score–matched cohort with a 1:1 ratio was constructed to adjust for baseline differences and to reduce confounding variables. Propensity scores were calculated involving the following preoperative and operative variables with significant intergroup differences: age, sex, SBP, SBP < 90 mmHg, intubation, hemopericardium, cardiac tamponade, malperfusion, axillary artery cannulation, entry tear excision, isolated AsAo replacement, arch replacement, partial arch replacement, total arch replacement, CPB time, aortic clamp time, circulatory arrest time, ACP, and RCP. After a propensity score-matching, a matched cohort of 56 patients per group was created. A multivariate logistic regression analysis was performed to identify the independent risk factors of in-hospital mortality. The variables for the multivariate logistic regression analysis were selected from those with a *P* < 0.05 in the univariate logistic regression analysis. The Kaplan–Meier method was used to estimate the 5-year cumulative survival and freedom from aortic reoperation rates of the two groups, which were compared using the log-rank test. For all analyses, statistical significance was set at *P* < 0.05.

## Results

### Patient demographics

As illustrated in Table 1, the D1 group was comprised of younger patients and fewer female patients than in the D2 group. In terms of preoperative conditions, preoperative shock (SBP < 90 mmHg; 21.5% vs 37.5%; *P* = 0.007) and intubation (5.3% vs 14.3%; *P* = 0.008) occurred less frequently in the D1 group. As for clinical presentation, the D1 group had lower rates of hemopericardium (29.7% vs 66.1%; *P* < 0.001) and cardiac tamponade (10.5% vs 30.4%; *P* < 0.001), but a higher rate of preoperative malperfusion (17.1% vs 5.4%; *P* = 0.022). All preoperative factors with significant intergroup differences were homogenized after propensity score-matching.

**Table 1:** Preoperative characteristics according to the patient group

Parameters	Overall cohort				Propensity-matched cohort			
	Total (n = 599)	D1 (n = 543)	D2 (n = 56)	*P* value	Total (n = 112)	D1 (n = 56)	D2(n = 56)	*P* value
Clinical demographics								
Age (years)	56.5 ± 13.6	55.3 ± 13.0	67.7 ± 14.5	< 0.001	65.3 ± 13.4	63.0 ± 11.8	67.7 ± 14.5	0.060
Sex (female, n, %)	185, 30.9	155, 28.5	30, 53.6	< 0.001	58, 51.8	28, 50.0	30, 53.6	0.705
Hypertension (n, %)	429, 71.6	389, 71.6	40, 71.4	0.973	76, 67.9	36, 64.3	40, 71.4	0.418
Diabetes mellitus (n, %)	39, 6.5	35, 6.4	4, 7.1	0.840	8, 7.1	4, 7.1	4, 7.1	0.999
Creatinine (mg/dL)	1.4 ± 1.4	1.4 ± 1.5	1.4 ± 0.8	0.697	1.4 ± 1.0	1.4 ± 1.1	1.4 ± 0.8	0.807
eGFR (mL/min/1.73 m^2^)	68.5 ± 30.3	68.6 ± 27.8	68.3 ± 48.8	0.967	64.5 ± 38.5	60.6 ± 23.9	68.3 ± 48.8	0.294
ESRD (n, %)	10, 1.7	10, 1.8	0	0.306	1, 0.9	1, 1.8	0	0.315
Preoperative condition								
SBP (mmHg)	97.2 ± 18.8	98.0 ± 17.8	90.1 ± 25.7	0.029	90.1 ± 22.7	90.0 ± 19.5	90.1 ± 25.7	0.987
SBP < 90 mmHg (n, %)	138, 23.0	117, 21.5	21, 37.5	0.007	41, 36.6	20, 35.7	21, 37.5	0.844
Intubation (n, %)	37, 6.2	29, 5.3	8, 14.3	0.008	13, 11.6	5, 8.9	8, 14.3	0.376
Time from ED to OR (h)	5.2 ± 4.5	5.1 ± 3.8	6.2 ± 9.2	0.369	5.8 ± 6.6	5.4 ± 1.7	6.2 ± 9.2	0.497
Repeat surgery (n, %)	21, 3.5	19, 3.5	2, 3.6	0.978	8, 7.1	6, 10.7	2, 3.6	0.142
Clinical presentation								
Chest/back pain (n, %)	446, 74.5	409, 75.3	37, 66.1	0.131	78, 69.6	41, 73.2	37, 66.1	0.411
Aortic regurgitation > moderate (n, %)	85, 14.2	77, 14.2	8, 14.3	0.983	22, 19.6	14, 25.0	8, 14.3	0.154
Hemopericardium (n, %)	198, 33.1	161, 29.7	37, 66.1	< 0.001	70, 62.5	33, 58.9	37, 66.1	0.435
Cardiac tamponade (n, %)	74, 12.4	57, 10.5	17, 30.4	< 0.001	30, 26.8	13, 23.2	17, 30.4	0.393
Acute myocardial infarction (n, %)	13, 2.2	10, 1.8	3, 5.4	0.086	4, 3.6	1, 1.8	3, 5.4	0.309
Malperfusion^a^ (n, %)	96, 16.0	93, 17.1	3, 5.4	0.022	6, 5.4	3, 5.4	3, 5.4	0.999
Intramural hematoma (n, %)	122, 18.7	101, 18.6	11, 19.6	0.849	18, 16.1	7, 12.5	11, 19.6	0.303

^a^Occurrence of preoperative limb ischemia, stroke, paraplegia, coronary artery occlusion, and mesenteric ischemia

D1, DeBakey type I; D2, DeBakey type II; ED, emergency department; eGFR, estimated glomerular filtration rate; ESRD, end-stage renal disease; OR, operating room; SBP, systolic blood pressure

### Surgical information

Table 2 showed detailed information regarding surgical variables. The femoral artery was the most commonly used vascular access of cannulation for > 95% of patients in both groups. More patients in the D1 group underwent additional axillary artery cannulation (88.6% vs 76.8%; *P* = 0.011) and the ACP strategy (90.2% vs 76.8%; *P* = 0.002) than in the D2 group. A higher rate of aortic arch replacement was observed in the D1 group (33.9% vs 5.4%; *P* < 0.001); however, the D1 group had a lower rate of entry tear excision (71.8% vs 96.4%; *P* < 0.001) compared to the D2 group. In the D2 group, 3 patients underwent 1/3 arch replacement with the innominate artery reimplantation because isolated AsAo replacement cannot be performed securely; one patient had extensive calcification of AsAo and proximal aortic arch and 2 had aneurysmal dilatation at distal AsAo. The time spans of CPB, aortic cross-clamping, and circulatory arrest were generally longer in the D1 group. All intraoperative factors with significant intergroup differences were homogenized after propensity score-matching.

**Table 2:** Surgical information according to patient group

Parameters	Overall cohort				Propensity-matched cohort			
	Total (n = 599)	D1 (n = 543)	D2 (n = 56)	*P* value	Total (n = 112)	D1 (n = 56)	D2 (n = 56)	*P* value
Femoral artery cannulation (n, %)	572, 95.5	517, 95.2	55, 98.2	0.302	111, 99.1	56, 100	55, 98.2	0.315
Axillary artery cannulation (n, %)	524, 87.5	481, 88.6	43, 76.8	0.011	84, 75.0	41, 73.2	43, 76.8	0.663
AsAo cannulation (n, %)	1, 0.2	1, 0.2	0	0.748	0	0	0	0.999
Aortic repair procedures								
Entry tear excision (n, %)	444, 74.1	390, 71.8	54, 96.4	< 0.001	107, 95.5	53, 94.6	54, 96.4	0.647
Root replacement (n, %)	65, 10.9	56, 10.3	9, 16.1	0.187	18, 16.1	9, 16.1	9, 16.1	0.999
Isolated AsAo replacement (n, %)	361, 60.3	316, 58.2	45, 80.4	0.001	89, 79.5	44, 78.6	45, 80.4	0.815
Arch replacement (n, %)	187, 31.2	184, 33.9	3, 5.4	< 0.001	6, 5.4	3, 5.4	3, 5.4	0.999
Partial arch (n, %)	125, 20.9	122, 22.5	3, 5.4	0.003	6, 5.4	3, 5.4	3, 5.4	0.999
Total arch (n, %)	62, 10.4	62, 11.4	0	0.008	0	0	0	0.999
Cardiopulmonary bypass time (min)	255.2 ± 79.6	257.8 ± 81.4	229.9 ± 53.6	0.001	238.7 ± 64.0	247.5 ± 72.5	229.9 ± 53.6	0.147
Aortic clamping time (min)	165.8 ± 55.6	167.2 ± 57.0	152.6 ± 37.6	0.011	157.8 ± 46.6	163.0 ± 54.0	152.6 ± 37.6	0.237
Circulatory arrest time (min)	50.9 ± 24.8	51.5 ± 25.5	45.1 ± 15.8	0.008	46.6 ± 18.9	48.2 ± 21.7	45.1 ± 15.8	0.396
ACP (n, %)	533, 89.0	490, 90.2	43, 76.8	0.002	83, 74.1	40, 71.4	43, 76.8	0.518
RCP (n, %)	66, 11.0	53, 9.8	13, 23.2	0.002	29, 25.9	16, 28.6	13, 23.2	0.518
Delayed sternum closure^a^ (n, %)	99, 16.5	92, 16.9	7, 12.5	0.394	13, 11.6	6, 10.7	7, 12.5	0.768
ECMO support (n, %)	21, 3.5	21, 3.9	0	0.134	0	0	0	0.999

ACP, antegrade cerebral perfusion; AsAo, ascending aorta; D1, DeBakey type I; D2, DeBakey type II; ECMO, extracorporeal membrane oxygenation; RCP, retrograde cerebral perfusion

^a^Mediastinal packing for uncontrolled hemorrhage and planned secondary exploration

### Postoperative complications

As Table 3 illustrates, the D1 group had a higher in-hospital mortality rate than the D2 group in the overall (15.8% vs 5.4%; *P* = 0.036) and propensity-matched cohorts (19.6% vs 5.4%; *P* = 0.022). In addition, postoperative malperfusion-related complications occurred more frequently in the D1 group before (24.3% vs 12.5%; *P* = 0.046) and after (30.4% vs 12.5%; *P* = 0.021) propensity-matching. A prolonged hospital stay in the D1 group was found in the overall cohort (25.6 ± 31.1 vs 19.0 ± 13.3 days; *P* = 0.004), but the statistical significance was not reached in the propensity-matched cohort (26.5 ± 26.5 vs 19.0 ± 13.3 days; *P* = 0.064).

**Table 3:** Postoperative mortality and morbidity according to patient group

Parameters	Overall cohort				Propensity-matched cohort			
	Total (n = 599)	D1 (n = 543)	D2 (n = 56)	*P* value	Total (n = 112)	D1 (n = 56)	D2 (n = 56)	*P* value
In-hospital mortality (n, %)	89, 14.9	86, 15.8	3, 5.4	0.036	14, 12.5	11, 19.6	3, 5.4	0.022
Bleeding (n, %)	16, 2.7	16, 2.9	0	0.193	1, 0.9	1, 1.8	0	0.315
Myocardial failure (n, %)	36, 6.0	35, 6.4	1, 1.8	0.162	6, 5.4	5, 8.9	1, 1.8	0.093
Brain stem failure (n, %)	21, 3.5	20, 3.7	1, 1.8	0.462	3, 2.7	2, 3.6	1, 1.8	0.558
Sepsis (n, %)	16, 2.7	15, 2.8	1, 1.8	0.666	4, 3.6	3, 5.4	1, 1.8	0.309
Re-exploration for bleeding (n, %)	92, 15.4	88, 16.2	4, 7.1	0.073	10, 8.9	6, 10.7	4, 7.1	0.508
Atrial fibrillation (n, %)	43, 7.2	37, 6.8	6, 10.7	0.282	11, 9.8	5, 8.9	6, 10.7	0.751
Delirium (n, %)	108, 18.0	102, 18.8	6, 10.7	0.135	17, 15.2	11, 19.6	6, 10.7	0.188
Seizure (n, %)	41, 6.8	37, 6.8	4, 7.1	0.926	9, 8.0	5, 8.9	4, 7.1	0.728
Brain stroke (n, %)	97, 16.2	92, 16.9	5, 8.9	0.121	16, 14.3	11, 19.6	5, 8.9	0.105
Infarction (n, %)	84, 14.5	82, 15.1	5, 8.9	0.212	15, 13.4	10, 17.9	5, 8.9	0.165
Hemorrhage (n, %)	19, 3.2	19, 3.5	0	0.155	1, 0.9	1, 1.8	0	0.315
Renal failure (n, %)	53, 8.8	50, 9.2	3, 5.4	0.334	9, 8.0	6, 10.7	3, 5.4	0.297
Mesenteric ischemia (n, %)	17, 2.8	17, 3.1	0	0.179	2, 1.8	2, 3.6	0	0.154
Limb ischemia (n, %)	18, 3.0	18, 3.3	0	0.167	2, 1.8	2, 3.6	0	0.154
Malperfusion-related complications^a^ (n, %)	139, 23.2	132, 24.3	7, 12.5	0.046	24, 21.4	17, 30.4	7, 12.5	0.021
Pneumonia (n, %)	75, 12.5	66, 12.2	9, 16.1	0.399	18, 16.1	9, 16.1	9, 16.1	0.999
Deep sternal wound infection (n, %)	17, 2.8	17, 3.1	0	0.179	3, 2.7	3, 5.4	0	0.079
ICU stay (days)	7.3 ± 14.5	7.4 ± 14.7	6.2 ± 11.7	0.560	6.1 ± 9.0	6.0 ± 5.1	6.2 ± 11.7	0.917
ICU readmission (n, %)	33, 5.5	30, 5.5	3, 5.4	0.958	6, 5.4	3, 5.4	3, 5.4	0.999
Hospital stay (days)	25.0 ± 29.9	25.6 ± 31.1	19.0 ± 13.3	0.004	22.7 ± 21.2	26.5 ± 26.5	19.0 ± 13.3	0.064

D1, DeBakey type I; D2, DeBakey type II; ICU, intensive care unit

^a^Occurrence of postoperative renal failure, brain infarction, mesenteric ischemia, and limb ischemia

### Risk factors associated with in-hospital mortality

Table 4 shows the logistic regression analyses results for in-hospital mortality in patients undergoing ATAAD repair based on the overall and propensity-matched cohorts, respectively. Eight independent risk factors for in-hospital mortality were identified in the overall cohort: D1-AAD, preoperative estimated glomerular filtration rate, preoperative intubation, prolonged CPB, aortic cross-clamping, and circulatory arrest times, RCP, and intraoperative ECMO support. Three independent risk factors for in-hospital mortality were identified in the propensity-matched cohort: D1-AAD, preoperative intubation, and RCP.

**Table 4:** Multivariate logistic regression analysis for in-hospital mortality

Parameters	β-coefficient	Standard error	Odds ratio, 95% CI	*P *value	
Overall cohort					
D1-AAD	1.756	0.713	5.79 (1.43–23.43)	0.014	
eGFR	− 0.013	0.006	0.98 (0.97–0.99)	0.021	
Intubation	1.203	0.449	3.33 (1.38–8.03)	0.007	
Cardiopulmonary bypass time	0.013	0.003	1.01 (1.01–1.02)	< 0.001	
Aortic clamping time	0.016	0.005	1.02 (1.01–1.03)	0.002	
Circulatory arrest time	0.019	0.007	1.02 (1.01–1.03)	0.010	
RCP	1.257	0.376	3.52 (1.68–7.35)	0.001	
ECMO support	1.806	0.597	6.08 (1.89–19.59)	0.002	
Propensity-matched cohort					
D1-AAD	2.588	0.955	13.31 (2.05–86.51)	0.007	
Intubation	2.935	0.905	18.82 (3.20–110.82)	0.001	
RCP	1.657	0.724	5.24 (1.27–21.69)	0.022	

D1-AAD, DeBakey type I acute aortic dissection; ECMO, extracorporeal membrane oxygenation; eGFR, estimated glomerular filtration rate; CI, confidence interval; RCP, retrograde cerebral perfusion

### Cumulative 5-year survival and freedom from reoperation rates

**Fig. 3:**
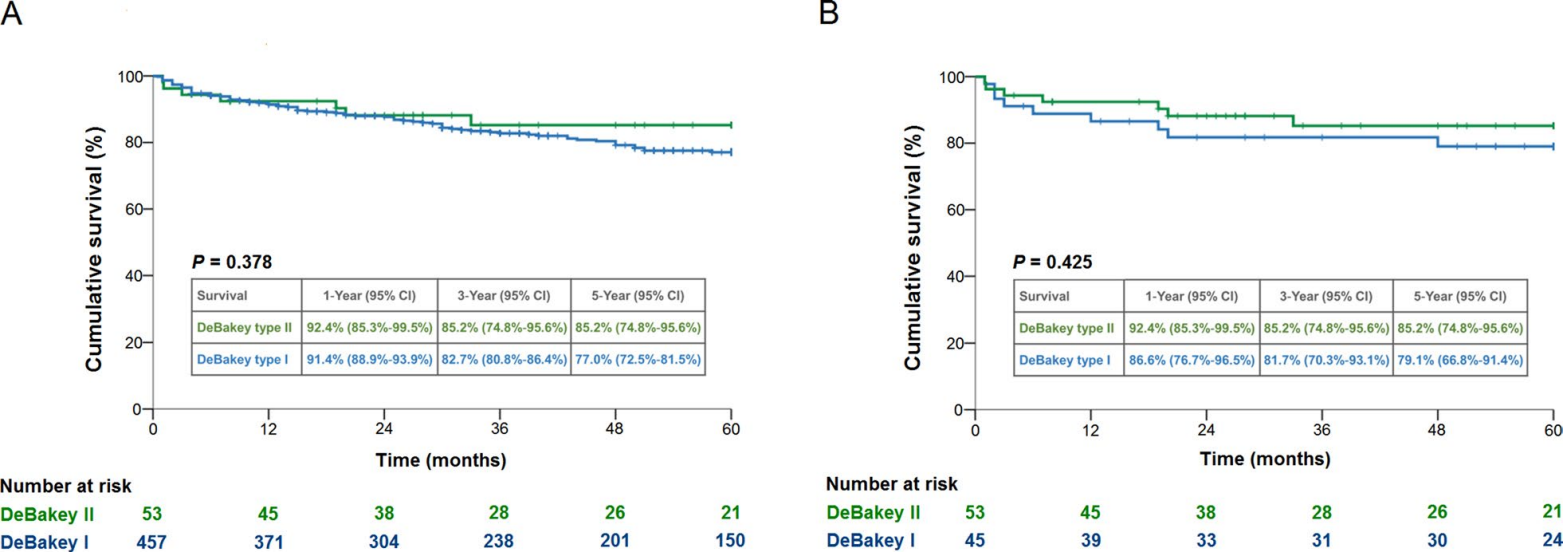
Five-year cumulative survival rates of the overall cohort (**a**) and propensity-matched cohort (**b**) stratified by DeBakey type I and type II

**Fig. 4:**
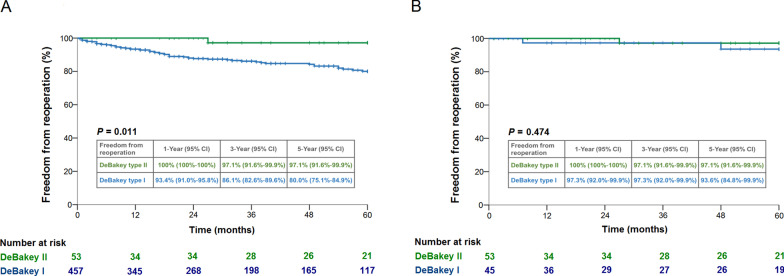
Five-year freedom from aortic reoperation rates of the overall cohort (**a**) and propensity-matched cohort (**b**) stratified by DeBakey type I and type II

Follow-up was completed for all patients with an average of 4.3 ± 3.4 years (median, 3.7; range, 0.1–11.8 years). As illustrated in Fig. 3, the 5-year cumulative survival rates for patients who survived to discharge were similar in D1 and D2 groups among the overall (77.0% vs 85.2%; *P* = 0.378) (Fig. 3a) and propensity-matched cohorts (79.1% vs 85.2%; *P* = 0.425) (Fig. 3b). In regard to the aspect of aortic reoperation, D1 group had a lower 5-year freedom from aortic reoperation rate compared to D2 group in the overall cohort (80.0% vs 97.1%; *P* = 0.011) (Fig. 4a). However, the freedom from aortic reoperation rates were similar for the two groups after propensity-matching (93.6% vs 97.1%; *P* = 0.474) (Fig. 4b).

## Discussion

Patients with D1-AAD were associated with inferior outcomes compared to those with D2-AAD in the previous literature [6, 7, 13]. However, great differences in preoperative and operative characteristics between D1-AAD and D2-AAD commonly exist because of the nature of different vascular anatomy. In this study, propensity score-matching was conducted to reduce the potential bias caused by confounding factors. For 599 patients who underwent aortic repair for ATAAD during the study period, 543 and 56 patients were classified as the D1 and D2 groups, respectively. A matched cohort of 56 patients per group was created. Regarding preoperative condition, higher risks of shock and cardiac tamponade in the D2 group and a higher risk of organ malperfusion in the D1 group were observed before propensity-matching. Furthermore, a more aggressive aortic arch repair strategy with, however, a lower rate of entry tear resection was observed in the D1 group. The D1 group had a significantly higher in-hospital mortality rate than the D2 group, and D1-AAD represented an independent risk factor for in-hospital mortality either before or after propensity-matching. In contrast, for patients who survived to hospital discharge, the D1 and D2 groups revealed similar 5-year survival rates in both the overall and propensity-matched cohorts.

Although all preoperative and intraoperative factors were homogenized by propensity-matching, the D1 group had higher rates of in-hospital mortality and malperfusion-related complications, which were consistent with the results of the overall cohort. There are several interpretations to our findings. First, organ malperfusion observed in AAD is correlated with its complex anatomic interactions between the true lumen and false lumen along the dissected aorta. These anatomic interactions can be dynamic during aortic repair surgery and are potentially affected by the surgical procedure itself. The dissected aortic segment in D2-AAD is limited to the AsAo and, theoretically, no residual dissection exists after aortic repair surgery. In contrast, complications may arise from the residual dissected arch and descending thoracic aorta in D1-AAD, especially when entry tear resection is not achieved and the false lumen remains patent. Furthermore, a dissected branch vessel without preoperative malperfusion symptoms may not maintain adequate perfusion to the supplied organ during CPB and circulatory arrest, which render a different circulation pattern compared to the normal physiology. Second, in the D1 group, approximately 30% of patients had postoperative malperfusion-related complications and > 50% (10/17) of these complications were brain infarctions. In the present study, the ACP strategy was used for only 74.1% of patients in the propensity-matched cohort. As reported by Perreas et al., ACP during the ascending and aortic arch surgical procedures, including ATAAD repair, is associated with a decreased risk of all types of neurological complications and trends toward decreased 30-day and mid-term mortality rates in comparison with RCP [14]. Therefore, we suggest that this modality may be applied more aggressively to reduce cerebrovascular complications, especially among patients with D1-AAD. Furthermore, compared to D1-AAD, extensive manipulation of the aortic arch is usually unnecessary during aortic repair of D2-AAD. This conservative manipulation of the aortic arch may reduce the potential risks of iatrogenic injury to branch vessels and related complications. In addition, the second main cause of postoperative malperfusion was acute renal failure. At this institute, if acute renal failure develops after ATAAD surgery, the renal replacement therapy is promptly implemented to reduce the negative impact of fluid overload, metabolic acidosis, and electrolyte imbalance according to the Acute Kidney Injury Network criteria [15]. However, in several studies, the occurrence of acute renal failure after ATAAD surgery revealed a major end-organ malperfusion, which indicated inadequate systemic perfusion and was correlated with inferior early and late survival [16, 17]. Therefore, once acute renal failure develops, the patient should be cautiously re-evaluated for hemodynamic targets, cardiopulmonary function perseverance, and systemic organ perfusion. Finally, among patients with branch vessel dissection with residual false lumen patency, a more aggressive strategy may also be applied for detecting and treating postoperative malperfusion-related complications early.

As reported by Kohl et al., D1-AAD and D2-AAD yielded similar late survival rates for patients who survived to index hospitalization discharge [13]. Similar outcomes were observed in the present study; patients who survived to discharge in the D1 and D2 groups had comparable 5-year survival rates in both of the overall cohort and propensity-matched cohort. In contrast, the D2 group had a higher freedom from aortic reoperation rate in the overall cohort, but this advantage was attenuated in the propensity-matched cohort. We suggest that this finding is highly correlated with the homogenized aortic repair procedures and entry tear excision rates. As reported by Inoue and Kimura et al., residual primary entry tears and patent false lumen were significant risk factors for aortic reoperation [18, 19]. Among the overall cohort, even with aggressive aortic arch replacement, the entry tear excision rate was only 71.8% in the D1 group, which was significantly lower than that in the D2 group (96.4%). Therefore, in addition to aortic arch replacement, hybrid endovascular stent-graft implantation to seal the entry tears located at the descending thoracic aorta may also be considered to reduce the dissection-related reoperation risks in carefully selected D1-AAD patients [20].

### Limitations

Several limitations to the present study should be clarified. First, as a retrospective and non-randomized control study, potential bias influencing the homogeneity of the D1 and D2 groups might have existed. However, with propensity score-matching, the intergroup heterogeneity was minimized. Furthermore, postoperative outcomes among the overall cohort and propensity-matched cohort were both well-presented and analyzed in this study. Second, because this crossed cohort spanned a period of nearly 12 years, the management regarding CPB and myocardial protection, as well as cerebral protection strategies and postoperative care protocols for treating ATAAD, may have evolved over time. Finally, despite the convincing early and late outcomes of the present study, an extended follow-up study should be conducted in the future to evaluate the long-term trends of D1-AAD and D2-AAD populations.

## Conclusions

D1-AAD was associated with higher risks of in-hospital mortality and postoperative malperfusion-related complications than D2-AAD. However, for patients who survived to hospital discharge, the late survival rates were comparable between patients with D1-AAD and D2-AAD during a 5-year follow-up.

## Data Availability

The datasets generated and analyzed during the current study cannot be made publicly available for ethical and legal reasons. The Institutional Review Board of Chang Gung Medical Foundation must review any request to share data publicly in order to protect patients' privacy. Requests for data can be sent to the Institutional Review Board of Chang Gung Medical Foundation at irb1@cgmh.org.tw.

## Abbreviations

AAD: Acute aortic dissection
ACP: Antegrade cerebral perfusion
AsAo: Ascending aorta
ATAAD: Acute type A aortic dissection
CI: Confidence interval
CPB: Cardiopulmonary bypass
D1: DeBakey type I
D2: DeBakey type II
ECMO: Extracorporeal membrane oxygenation
ED: Emergency department
eGFR: Estimated glomerular filtration rate
ESRD: End-stage renal disease
ICU: Intensive care unit
OR: Operating room
RCP: Retrograde cerebral perfusion
SBP: Systolic blood pressure
